# Expression of CD40 and CD192 in Classical Monocytes in Multiple Sclerosis Patients Assessed with Transcranial Magnetic Stimulation

**DOI:** 10.3390/biomedicines11102870

**Published:** 2023-10-23

**Authors:** Nikolina Režić Mužinić, Anita Markotić, Sanda Pavelin, Denis Polančec, Maja Buljubašić Šoda, Antonia Bralić, Joško Šoda, Angela Mastelić, Una Mikac, Ana Jerković, Maja Rogić Vidaković

**Affiliations:** 1 Department of Medical Chemistry and Biochemistry, School of Medicine, University of Split, 21000 Split, Croatia; anita.markotic@mefst.hr (A.M.);; 2 Department of Neurology, University Hospital of Split, 21000 Split, Croatia; 3 CytomEx, 10000 Zagreb, Croatia; 4 Department of Pediatrics, University Hospital of Split, 21000 Split, Croatia; 5 Department of Interventional and Diagnostic Radiology, University Hospital of Split, 21000 Split, Croatia; 6 Signal Processing, Analysis, Advanced Diagnostics Research and Education Laboratory (SPAADREL), Department for Marine Electrical Engineering and Information Technologies, Faculty of Maritime Studies, University of Split, 21000 Split, Croatia; 7 Department of Psychology, Faculty of Humanities and Social Sciences, University of Zagreb, 10000 Zagreb, Croatia; 8 Laboratory for Human and Experimental Neurophysiology, Department of Neuroscience, School of Medicine, University of Split, 21000 Split, Croatia

**Keywords:** multiple sclerosis, CD40, CD192, CD14^++^CD16^−^ monocytes, CD14^+^CD16^++^ monocytes, transcranial magnetic stimulation, TMS, motor-evoked potentials, MEP, corticospinal tract

## Abstract

Expression of CD40 and CD192 markers in different monocyte subpopulations has been reported to be altered in people with MS (pwMS). Also, functional connectivity of the corticospinal motor system pathway alterations has been proved by transcranial magnetic stimulation (TMS). The study objective was to investigate the expression of CD40 and CD192 in classical (CD14^++^CD16^−^), intermediate CD14^++^CD16^+^ and non-classical (CD14^+^CD16^++^) blood monocyte subpopulations in pwMS, undergoing neurophysiological TMS assessment of the corticospinal tract integrity by recording motor-evoked potentials (MEPs). Radiological examination on lesion detection with MRI was performed for 23 patients with relapsing–remitting MS treated with teriflunomide. Then, immunological analysis was conducted on peripheral blood samples collected from the patients and 10 healthy controls (HC). The blood samples were incubated with anti-human CD14, CD16, CD40 and CD192 antibodies. Next, pwMS underwent neurological testing of functional disability (EDSS) and TMS assessment with recording MEPs from upper and lower extremity muscles. The results show that in comparison to HC subjects, both pwMS with normal and altered MEP findings (prolonged MEP latency or absent MEP response) had significantly decreased surface receptor expression measured (MFIs) of CD192 and increased CD40 MFI in classical monocytes, and significantly increased percentages of classical and total monocytes positive for CD40. Knowing CD40’s pro-inflammatory action, and CD192 as a molecule that enables the passing of monocytes into the brain, decreased CD192 in classical monocytes could represent a beneficial anti-inflammatory parameter.

## 1. Introduction

Multiple sclerosis (MS) is an inflammatory autoimmune disease of the central nervous system (CNS) that is still of unknown cause and characterized by demyelinating white matter lesions and neuronal degeneration, which causes various symptoms (motor and sensory dysfunctions, cognitive impairment, mood disorders, and fatigue) [1]. The prevalence of MS in the world ranges from 5 to 300 per 100,000 people and affects women three times more than men [2]. Relapsing–remitting MS (RRMS) is the most common form of the disease with a prevalence of about 80 to 90% of people with MS (pwMS). The primary progressive form of the disease (PPMS) is significantly less common and occurs in 10% of patients, while the further progression of the disease indicates the transition from relapsing–remitting to the secondary-progressive form of the disease (SPMS). The primary pathological event of MS is demyelination with degeneration and loss of axons, which results in a permanent functional deficit [2,3,4].

The diagnosis of MS is made according to the 2017 McDonald criteria and the 2021 MAGNIMS-CMSC-NAIMS recommendations (number and localization of lesions on the 1.5 T MRI, oligoclonal bands in the cerebrospinal fluid, and clinical picture) [5,6]. The clinical status of disability is expressed through the Expanded Disability Status Scale (EDSS) [7]. The key functional components of the EDSS scale, correlating with sustained disability progression, reflect mostly the status of the pyramidal (corticospinal) motor system [8]. Further, various quantitative radiological measures, derived from conventional and advanced MRI methods, have been proposed as prognostic biomarkers for MS. However, correlations between different MRI indicators and EDSS are not satisfactory, and no MRI measure is used as a comprehensive prognostic imaging biomarker for MS [9,10,11]. The treatment of MS depends on the specific clinical symptoms of the patient and may include treatment of relapse symptoms with steroid drugs (i.e., methylprednisolone) and treatment with the aim of reducing the number of relapses with disease-modifying drugs (DMD) such as teriflunomide, interferon beta 1a, interferon beta 1b, glatiramer acetate, fingolimod, alemtuzumab, cladribine, siponimod, ocrelizumab, peginterferon beta 1a, dimethyl fumarate, and natalizumab. Immunomodulatory drugs can suppress inflammation but cannot stop the inflammatory reaction in the CNS since neither the trigger of inflammation nor the specific target antigen of the CNS is yet known [3]. Since the pathogenesis of MS is a consequence of abnormal immune activation, the primary role of disease-modifying therapy is to limit the abnormal immune responses without compromising protective immune competence [12].

Immunological investigations demonstrated that MS is mediated by activated T cells with evidence of a significant contribution from B cells and cells of the innate immune system [13]. Monocytes, together with Th1 and Th17 cells, play a key role in CNS inflammation in MS [14]. Monocytes are often divided into three subpopulations depending on the expression of CD14 and CD16 (non-classical CD14^+^CD16^++^, intermediate CD14^++^CD16^+^ and classical CD14^++^CD16^−^) [15]. Previous studies reported downregulation in median CD40 and CD192 expression levels on different monocyte populations in MS disease compared to HC subjects [15].

The recent therapeutic strategies involve the depletion of peripheral blood cell populations (B cells using rituximab and ocrelizumab) or prevent T cells from entering into the CNS (natalizumab). A similar strategy targeting monocytes may also have potential in MS therapy [15]. Pathophysiological correlations with clinical findings and symptoms are still not fully elucidated, which suggests the need for more precise analyses and the discovery of immunological markers and other subclinical markers that could identify the pathological events involved with MS in different stages of the disease. Newly discovered parameters would allow treatment optimization with immunomodulating drugs, as well as monitoring the evolution of the disease. For example, subtle changes could be detected before clinically visible progression [16,17]. Experimental models have shown that inflammation induces synaptic hyperexcitability, and TMS studies have confirmed an imbalance between excitatory and inhibitory transmission in MS patients. Hence, inflammation-driven synaptic hyperexcitability could be present with distinct intensity in different phases of the MS disease course [16].

Most of the clinical symptoms, typical of MS, are related to the altered generation and transmission of signals in the CNS. Pathological signaling can result from various mechanisms including demyelination or a localized block in impulse conduction due to axonal damage [18]. Motor-evoked potentials (MEPs) represent neurophysiological measures of signal conduction in the CNS in vivo. Examining corticospinal excitability as a marker of functional integrity of the primary motor cortex (M1) and the corticospinal pathway using e-filed navigated transcranial magnetic stimulation (TMS) by assessing MEP could elucidate the underlying pathophysiological mechanisms of MS [16,17,19,20,21].

The present study aimed to combine the immunological investigation of the monocyte subpopulations with the neurophysiological investigation of the functional status of the pyramidal (corticospinal) tract integrity [22]. The study objective was to investigate the expression of CD40 and CD192 markers on the classical (CD14^++^CD16^−^), intermediate CD14^++^CD16^+^ and non-classical (CD14^+^CD16^++^) peripheral blood (PB) monocyte subpopulations of pwMS undergoing functional neurophysiological assessment of the corticospinal tract integrity via motor-evoked potential (MEP) recording.

## 2. Materials and Methods

### 2.1. Participants

Forty-six RRMS patients treated with teriflunomide (Aubagio; Sanofi, Tours, France) medication for ≥12 months were considered for inclusion in the study (University Hospital of Split, Split, Croatia). Eleven of them did not fulfill the inclusion criteria regarding the drug duration intake, and twelve refused to participate in the study (due to, e.g., living on islands, having problems with walking, etc.). The final study sample was composed of those who met the inclusion criteria and agreed to participate in the study and included twenty-three RRMS (relapsing–remitting MS) patients with a mean age of 41.65 ± 8.89 and ten HCs with a mean age of 37 ± 13.9. Most pwMS were women (60.87%), right-handed (91.3%), and had high school education (73.9%). The mean disease duration was 9.39 ± 5.73 years, and the median EDSS score was 2.5 (3.0). Out of 23 pwMS, 57.8% received corticosteroid treatment three or more than three times during the entire medical treatment. Teriflunomide was administered as indicated in the Aubagio product monograph (14 mg per day). Additionally, none of the pwMS experienced a relapse 3 months prior to participation in this study. HC samples from previous studies were included as referent values for MEP assessment for comparison with pwMS [23,24,25,26,27,28]. The following exclusion criteria for TMS assessment were applied: history of diseases of the central or peripheral nervous system other than RRMS, history of psychiatric diseases, presence of any contraindication for TMS, and drug or alcohol abuse [29]. Table 1 presents the basic characteristics of pwMS and HC subjects for the immunological analysis.

### 2.2. The Data Collection Procedures: PB Collection, Flow Cytometry, Clinical Assessment (Neurologic and Radiologic), and TMS Examination

**Figure 1:**
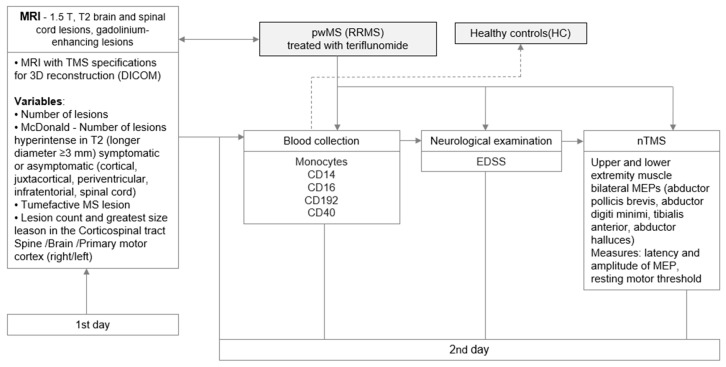
The data collection procedure. The clinical parameters assessed in the 1st day include radiological magnetic resonance imaging (MRI) for lesion detection. The examinations assessed on the 2nd day include: PB collection and immunological investigation of monocyte subpopulation markers, neurological evaluation applying Expanded Disability Status Scale (EDSS), and e-field navigated transcranial magnetic stimulation (nTMS) assessing the integrity of corticospinal tract by recording motor-evoked potentials (MEPs) from upper and lower extremity muscles.

First, radiological MRI assessment of the brain and spinal cord was conducted. One or two days later, PB was collected followed by neurological examination and TMS assessment on the same day. Collection of PB and neurological examination was performed at the University Hospital of Split (Department of Neurology), while TMS examination was performed at the University of Split School of Medicine (Department of Neuroscience). PB analyses were conducted at the University of Split School of Medicine (Department of Medical Chemistry Biochemistry). The diagram in Figure 1 illustrates the procedure of data collection for pwMS.

### 2.3. Flow Cytometry

In total, 100 µL of blood sample was incubated for 20 min in the dark at 25 °C with 4 µL of anti-human-CD14 FITC antibodies (BD Pharmingen, San Diego, CA, USA), 4 µL of phycoerythrin-conjugated antibodies reactive to human CD16 (BD Pharmingen, San Diego, CA, USA), 3 µL of mouse antibodies reactive to human CD192 conjugated with BB700 (BD Horizon, San Diego, CA, USA) and 5 µL of Alexa Flour 647 conjugated antibodies reactive to human CD40 (BD Pharmingen). Following the red blood cell lysis with BD Pharm Lyse^TM^ solution (BD Biosciences, San Diego, CA, USA), acquisition of samples was performed using a BD Accuri C6 (BD Biosciences, Aalst, Belgium) flow cytometer. Fluorochrome and isotype-matched controls as well as unstained cell samples were measured and processed as negative controls to set the appropriate regions. Cell acquisition was stopped at 10^6^ cells. Flow cytometry data for each marker were collected in one flow run.

#### Flow Cytometry Data Analysis

**Figure 2:**
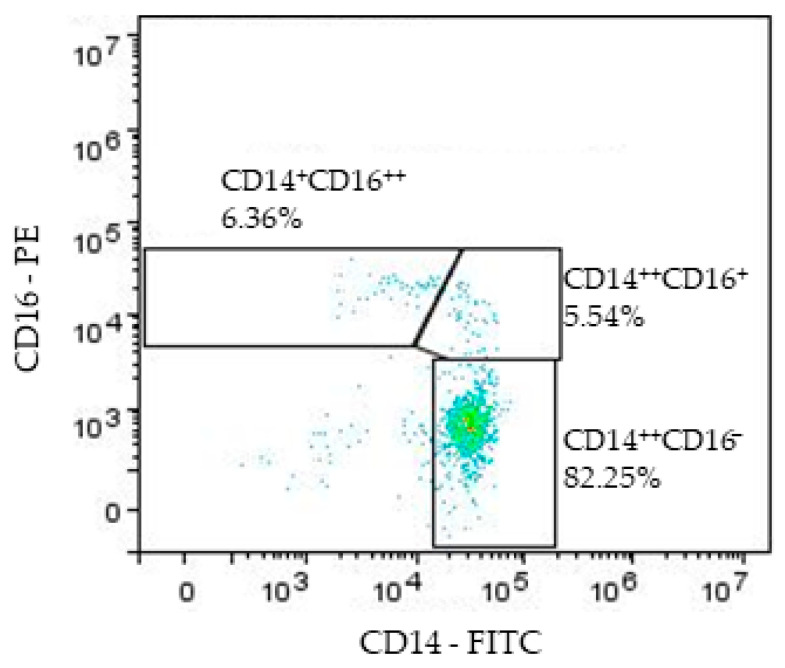
Representative gates for monocyte subpopulations: non-classical (CD14^+^CD16^++^); intermediate (CD14^++^CD16^+^); and classical (CD14^++^CD16^−^).

Data acquired by flow cytometer were analyzed using the FlowLogic Software version 6 (Inivai Technologies, Mentone Victoria, Australia). Monocytes were recognized in the forward scatter/side scatter (FSC/SSC) density plots. The FSC parameter correlates with cell diameter and SSC correlates with cell granularity. Gating strategies for monocyte subpopulations are shown in Figure 2. After the exclusion of doublets, the remaining cell population expressions of CD14 and CD16 were displayed in a CD16/CD14 plot to identify monocyte subsets. Further, the gated monocyte subpopulation was analyzed for its percentage and surface receptor expression (measured as MFI) of CD40 and CD192 in diagram CD40-Alexa Fluor 647 vs. CD192-BB700.

### 2.4. Clinical Examination and Corticospinal Excitability Investigation with Transcranial Magnetic Stimulation (TMS)

The clinical assessment consisted of neurological and radiological examinations. The neurological examination included demographic and multiple sclerosis data: age, gender, duration of MS diseases, and EDSS score. Radiological MRI assessment and image evaluation scans were acquired on a 1.5 T MR system (Avanto, Siemens, Medical Systems, Best, Germany) using a 12-channel phased array head coil. The sequences in the brain scan protocol were 3D T1-weighted images, axial T2-weighted images, and fluid-attenuated inversion recovery (FLAIR) images in the axial and sagittal plane. Spinal cord sequences included sagittal T2-weighted images, sagittal turbo inversion recovery magnitude (TIRM) images, and axial T2 me2d images from C1 to C7 vertebral levels. Using the T2, FLAIR, TIRM, and T2 me2d images, potential lesion locations of the corticospinal tract (CST) were visually examined, including subcortical white matter in the primary motor cortex (CST-M1), capsula interna, cerebral peduncles and ventral parts of the midbrain and pons (CST-M2) and ventral and lateral parts of the cervical spinal cord (CST-M3). The McDonald criteria were consulted for the lesion count for the individual subject [5,6]. MRI images were used for the 3D reconstruction of individual brain anatomy for TMS examination.

Neurophysiological TMS examination was performed using the e-field navigated system from Nexstim NBS System 4 (Nexstim Plc., Helsinki, Finland) for testing functional integrity of the corticospinal pathway by stimulating the M1 and recording MEPs from the muscles of the upper and lower extremities. MEPs were elicited by TMS with a biphasic magnetic coil generating a 289 µs pulse. A figure-of-eight coil with an inner coil diameter of 50 mm and an outer coil diameter of 70 mm was placed tangentially on the subject’s head at the level of the M1 for upper and lower limb muscles. The maximum electric field is 172 V/m under the TMS coil in a spherical conductor model representing the human head. MEPs were recorded from the muscles of the upper extremities (abductor pollicis brevis—APB, abductor digiti minimi—ADM) and muscles of the lower extremities (tibialis anterior—TA, abductor hallucis—AH) with a pair of self-adhesive surface electrodes (Ambu^®^ Blue Sensor BR, BR-50-K/12 manufactured by Ambu A/S, Ballerup, Denmark). The electrodes were connected by cable to the Nexstim electromyography (EMG) system with a 1.5 mm touch-resistant safety connector (DIN 42-802) to the six-channel EMG and common ground. The coil was placed tangentially to the central sulcus to ensure posterior-anterior current direction in the brain. The magnetic stimulation intensity in the present study was defined as the highest transcranial stimulus intensity at which TMS of the motor cortex produces reproducible EMG (MEP) response in the ‘target’ muscle. The 120% resting motor threshold intensity could not be properly applied, since in some patients, 100% of maximal stimulator output was required to elicit MEP response [22].

### 2.5. Statistical Analysis

Since parameters of skewness and kurtosis did not indicate great deviations from a normal distribution for most variables, the parametric statistic was used, except for EDSS for which we used nonparametric statistics (stated in brackets). Participants’ characteristics were assessed using descriptive statistics. Groups were compared with Welch *t*-tests suitable for heterogeneous variances (Mann–Whitney U test). These included comparisons of pwMS to healthy control samples from previous research [23,24,25,26,27,28], pwMS with altered TMS (MEP) findings (prolonged MEP latency or absent MEP), and pwMS with normal/regular TMS (MEP) findings. MEP latency and amplitude estimation was performed by a custom-made Matlab script (R2021a) using an automatic algorithm [30]. Correspondence in classification was tested with McNemar’s test. A *p* < 0.05 was considered statistically significant. Statistical analyses were conducted using Excel 2013 and IBM SPSS Statistics Version 25.

## 3. Results

### 3.1. Clinical and TMS Results of pwMS

According to McDonald’s evaluation, MRI analysis showed that the mean number of the lesions was 26.73 ± 20.54, while a total of 2.04 ± 1.79 lesions in the corticospinal tract (1 ± 1.08 lesion on the right, and 1.08 ± 1.04 on the left side) were detected. Fifteen pwMS (65.2%) had altered MEP findings with MEP latency prolonged or absent (with evident corticospinal tract lesion), while eight (34.8%) subjects had normal MEP findings [22]. From these eight subjects with normal MEP findings, only one MS patient had a lesion in the corticospinal tract.

### 3.2. Flow Cytometry Results of pwMS and HC Group

**Figure 3:**
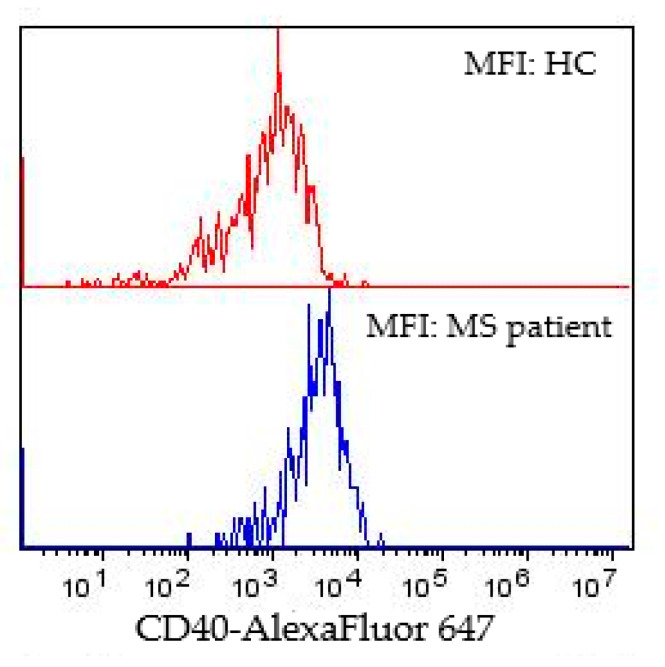
MFI of CD40 in classical monocytes of HC and MS patient. *p* < 0.05. Abbreviations: HC—healthy control; MS—multiple sclerosis.

All pwMS and pwMS with altered MEP findings differed from the HC in the percentage of CD40 in classical CD14^++^CD16^−^ monocytes, surface expression of CD40 and CD192 in classical CD14^++^CD16^−^ monocytes, and percentage of total monocytes positive for CD40 (Table 2). All pwMS and pwMS with altered MEP findings had significantly decreased surface expression of CD192 in classical monocytes (*p* < 0.01; and *p* < 0.001), while their CD40 surface expression was increased (both *p* < 0.05) as well as the percentage of CD40^+^ classical monocytes (*p* < 0.001; *p* < 0.01) and the percentage of CD40^+^ (*p* < 0.01; *p* < 0.05) in total monocytes (Table 2). Further, pwMS with normal MEP findings also had significantly decreased surface expression of CD192 classical monocytes (*p* < 0.05), significantly increased surface expression of CD40 (*p* < 0.05) and percentage of classical monocytes (*p* < 0.01), and significantly increased percentage of total monocytes positive for CD40 (*p* < 0.05) in comparison to HCs (Table 2). Figure 3 represents the surface expression of CD40 in classical monocytes in a single HC and MS patient. Maximal surface expression of CD40 was higher in MS patients (2 × 10^4^) than in the HC subjects (8 × 10^3^) (Figure 3). No significant difference was found for intermediate CD14^++^CD16^+^ monocytes between the groups examined. 

**Figure 4:**
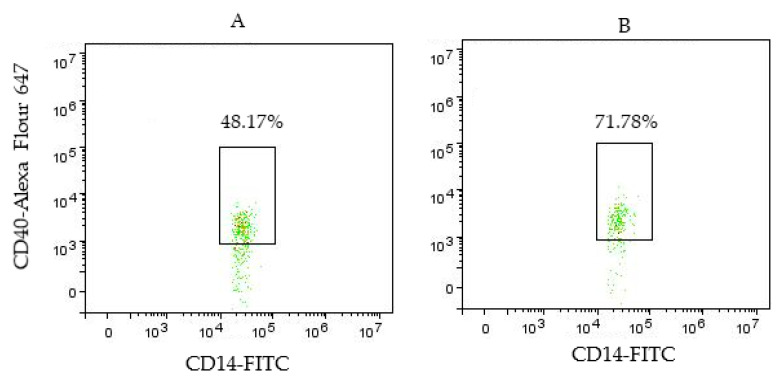
Comparison of the percentage of CD40^+^ classical monocytes of HC (**A**) and MS patient (**B**). *p* < 0.001. Abbreviations: HC—healthy control; MS—multiple sclerosis.

Figure 4 shows that percentage of CD40^+^ classical monocytes was increased in MS patients at 71.78% compared to 48.17% in HC.

**Figure 5:**
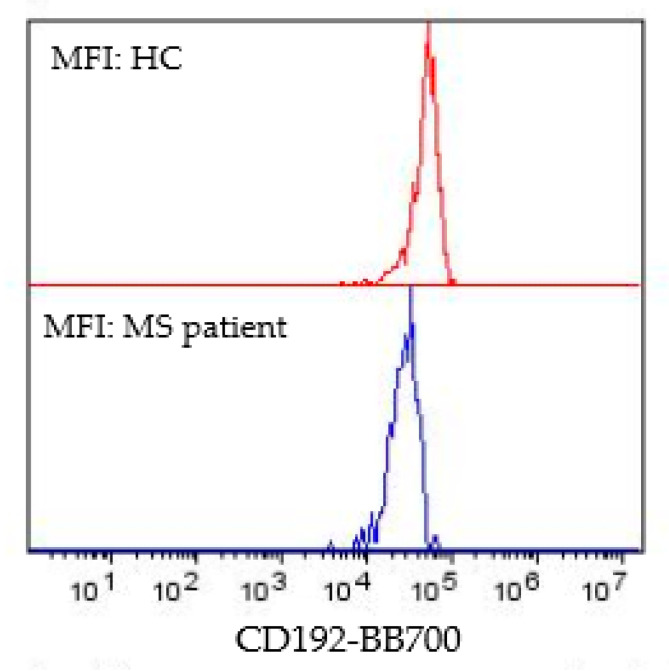
MFI of CD192 classical monocytes in HC and MS patients with altered MEP finding. *p* < 0.0001. Abbreviations: HC—healthy control; MS—multiple sclerosis; MEP—motor-evoked potentials.

Examples of the MFI of CD192^+^ classical monocyte in Figure 5 show decreased MFI in an MS patient with altered MEP finding, compared to the HC subject.

## 4. Discussion

This study represents the first attempt to apply the neurophysiological technique in evaluating corticospinal tract integrity combined with the immunological investigation of the expression of CD40 and CD192 markers in the classical (CD14^++^CD16^−^), intermediate (CD14^++^CD16^+^) and non-classical (CD14^+^CD16^++^) PB monocyte subpopulations of pwMS. The expression of CD192 in classical monocytes was significantly decreased in pwMS with altered MEP findings, and in pwMS with normal MEP findings in comparison to HC subjects. The percentage of total monocytes positive for CD40 and the expression of CD40 (expressed as MFI) in classical monocytes were significantly increased, both in pwMS with altered MEP findings and pwMS with normal MEP findings. No significant difference was found for non-classical and intermediate monocytes between the groups examined.

**Figure 6:**
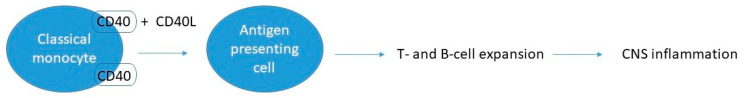
The role of monocyte CD40 in CNS inflammation. CD40 from the surface of classical monocytes binds to the CD40L, causing conversion of classical monocytes into antigen-presenting cells.

The pwMS in the present study received a single immunomodulatory medication, teriflunomide, which is known to decrease both inflammatory M1 markers (tumor necrosis factor α, TNF-α, and interleukin-6, IL-6) and anti-inflammatory M2 marker (Arginase-1, Arg1) in a time-dependent manner [31,32]. During autoimmune inflammation, Arg1 is upregulated in monocyte-derived cells due to their interaction with TNF-α activated blood–brain barrier cells [33]. Further, co-stimulating CD40 molecule is present in MS on different cell types, including B cells, monocytes, macrophages, endothelial cells, T cells, and CNS-resident cells [34,35]. Activation of these cells by CD40L (ligand) results in the secretion of pro-inflammatory cytokines and chemokines [36,37], and T cell expansion [38], which promotes the ongoing inflammation in the CNS [36,38]. The role of monocyte CD40 in CNS inflammation development is shown in Figure 6. Gjelstrup et al. [15] showed downregulation in median expression levels of CD40 and CD192 markers over the total monocyte population, in patients with a broad spectrum of MS disease, and both treated and untreated, compared to healthy controls. This finding was explained by an expanded non-classical monocyte population in pwMS, with only half of them expressing CD40 and 3% expressing CD192, compared to classical monocytes, which were almost 100% CD40^+^ and CD192^+^ [15,39]. It is known that the fraction of non-classical CD14^+^CD16^++^ monocytes is normally 10% of total monocytes, while major classical inflammatory monocytes CD14^++^CD16^−^ represent 85% of total monocytes and the rest belongs to the intermediate CD14^++^CD16^+^ (5%) monocyte subpopulation [40]. The diversity of monocyte subpopulations has been shown in the pathogenesis of inflammation, whereas a changed part of a specific population may co-exist as a biomarker of the disease [41]. Classical monocytes express high levels of CD192 allowing them to respond to injury [42], whilst non-classical monocytes act to patrol tissue endothelium and promote neutrophil adhesion [43]. Also, high monocyte CD192 expression is a precondition for their crossing the blood–CNS barrier [44]. Inflammation develops by monocyte conversion to antigen-presenting cells and co-stimulation leading to T and B cell activation and differentiation via the CD40 [45].

Classical inflammatory CD14^++^CD16^−^ monocytes, with high CD192 (also known as CCR2, a chemokine receptor that binds monocyte chemoattractant proteins, MCP-1) expression, due to interleukin-1β (IL-1β) secretion cross the blood–CNS barrier before experimental autoimmune encephalomyelitis onset [44]. Inside the CNS, blood-derived monocytes are converted into an antigen-presenting cell phenotype under the influence of IL-1β–induced secretion of encephalitogenic cytokine GM-CSF (granulocyte-macrophage colony-stimulating factor) by endothelial cells and then associate with proliferating CD4^+^ T cells. Factors released from the interaction between activated monocytes and CD4^+^ T cells are highly toxic to neurons [44]. The mice that received teriflunomide had lower protein levels of IL-1β [46].

Many disease-modifying treatments have shown different levels of efficacy in preventing relapses, and accumulation of lesions, but no MS cure is yet available [3]. Teriflunomide is a malononitrilamide, and its mechanism of action is based on selective and reversible inhibition of dihydro-orotate dehydrogenase (DHODH) [47]. Li et al. [32] showed that teriflunomide inhibits the release of MCP-1 (CCL2) in human monocytes stimulated by lipopolysaccharide (LPS) in a DHODH-independent manner. MCP-1 and its receptor, CCR2 (CD192), are involved in the recruitment of inflammatory cells (monocytes and T cells) into the CNS [48] and play a pro-inflammatory role in MS. Therefore, the inhibitory effect of teriflunomide on MCP-1 production could potentially result in decreased immune cell migration into the CNS [32]. Mice devoid of CCR2 show a marked reduction in monocyte extravasation [49] whereas mice lacking CCL2 show attenuated demyelination during experimental autoimmune encephalomyelitis [50]. The significance of CCL2 and CCR2 in MS is enigmatic because CCL2 levels are consistently decreased in the cerebrospinal fluid of pwMS despite abundant expression within lesioned multiple sclerosis tissues [51]. Mahad et al. [51] hypothesized that CCL2 is consumed by migrating inflammatory cells, which downregulate CCR2, as they cross the blood–brain barrier.

In this study, the percentage of total monocytes positive for CD40 and the expression of CD40 (expressed as MFI) in classical monocytes were significantly increased, both in pwMS with altered MEP findings and pwMS with normal MEP findings. Figure 6 depicts the role of monocyte CD40 in CNS inflammation. Contrary to our study results regarding CD40, Gjelstrup et al. [15] found a decreased percentage of CD40^+^ total monocytes in pwMS which was explained by the increased fraction of non-classical monocyte subpopulation in their sample containing a halved percent of CD40^+^ non-classical in relation to classical monocytes that all bear CD40.

Further, the present study found a significantly decreased expression of CD192 in classical monocytes in pwMS with altered MEP findings and pwMS with normal MEP findings in comparison to HC subjects. The percentage of non-classical monocytes was decreased in ex vivo analyses of monocytes from pwMS and showed significantly elevated CD192 levels after LPS stimulation especially on CD16^+^ monocytes (non-classical and intermediate monocytes) [52]. Due to the induction of inflammation with LPS stimulation, a decrease in CD192 is expected during pwMS treatment. This was proved by Cui et al. [53] who analyzed the percentage of each CD192^+^ monocyte subpopulation after IFN-β treatment. The results of the present study and the results of Cui et al. [53] for non-classical CD14^+^CD16^++^ monocytes are not fully comparable due to different descriptions of the same subpopulation as CD14^low^CD16^+^ but the percentage of non-classical CD192^+^CD14^low^CD16^+^ monocytes was lower, as in the present study, also without statistical significance [53]. On the other hand, IFN-β treatment decreased the percentage of CD192 positive monocytes in pwMS, designated as CD14^+^CD16^−^ (in our study marked as CD14^++^CD16^−^) [53]. Surface expression of CD192 is down-modulated even in the remission phase in MS, and down-modulation is rather enhanced by IFN-β therapy probably being one of the self-protective mechanisms against MS partly contributing to sustained remission by preventing monocyte infiltration into the CNS [53]. Positive co-stimulation of T cells in CNS is mediated by CD40^+^ macrophages, not monocytes, while monocyte programmed death-ligand 1 (PD-L1) is a costimulatory molecule that delivers a negative signal for T cell activation [45]. Due to the decreased expression of CD192 and increased expression of CD40 in classical monocytes, in spite of the potential pro-inflammatory effect of elevated CD40 at macrophages, which is expected to be formed within CNS, less CD192 expression per monocyte could result in lower passing of the blood–CNS barrier. The particular feature of teriflunomide is to induce monocyte PD-L1, a molecule involved in tolerance to autoantigens, which can contribute to the inhibition of the abnormal immune response in MS [54].

Subjects in this study were mildly disabled (according to clinical EDDS findings), but we graded them as those with normal and altered MEP findings according to our recently published study [55]. In a previously published study, we investigated TMS measures (resting motor threshold (RMT), motor-evoked potential (MEP) latency, and MEP amplitude) of corticospinal tract integrity in pwMS. pwMS with altered MEP latency (prolonged or absent MEP response) had higher EDSS general and higher EDSS pyramidal functional scores than MS people with normal MEP latency findings. TMS/MEP latency findings classified people with MS as the same as EDSS functional pyramidal scores in 70–83% of cases and were similar to the MRI results, corresponding to EDSS functional pyramidal scores in 57–65% of cases. pwMS with altered MEP latency differed from people with MS with normal MEP latency in the total number of lesions in the brain corticospinal and cervical corticospinal tract. Therefore, the study provided preliminary results on the correspondence of MRI and TMS corticospinal tract evaluation by inspecting MEP latency results with EDSS functional pyramidal score results in MS [55]. Therefore, according to the findings of MFI of CD192 and CD40 in CD14^++^CD16^−^ monocytes in two MS subgroups, we can speculate about possible findings in more disabled patients. CD192 expression in both MS groups was decreased (19% lower in pwMS with altered MEP latency findings and 13% lower in pwMS with normal MEP latency findings) while their CD40 expression was increased (65% higher in pwMS with altered MEP latency findings and 54% higher in pwMS with normal MEP latency findings) in comparison to healthy control. Therefore, more disabled patients could further mildly decrease CD192 expression and significantly increase CD40 expression accompanied by more prolonged or absent MEP response.

This study has several limitations. It comprises a relatively small number of pwMS as was also noticed in previous studies [56], and longitudinal follow-up is also needed to gain more insights on the clinical relevance of TMS assessment of corticospinal tract integrity with investigation of the expression of CD40 and CD192 in classical monocytes in MS. Furthermore, the present study investigated only monocytes. Lymphocyte analysis could be a promising direction for future study, due to the fact that inhibition of DHODH by teriflunomide causes cell cycle arrest at the S phase and has a cytostatic effect on the proliferation of activated (T and B) lymphocytes, which are responsible for the inflammatory process of MS [57].

The finding of decreased expression of CD192 in classical monocytes in pwMS deserves its following. Future research should apply longitudinal monitoring of the functional and immunological status, which might provide subclinical MS disease-related information in addition to the standard clinical MS status. In addition to monocyte investigation, determination of TMS parameters MEPs in relation to B and T cell markers in patients treated with rituximab/ocrelizumab and natalizumab, respectively, is worthy of performing. Due to defined therapy targets, more specific correlations between some B or T cell markers and MEPs in patients in different MS stages are expected.

## Figures and Tables

**Table 1:** Comparison of basic characteristics of pwMS and HC group.

Parameter Mean ± SD	pwMS (*n* = 23)	HC (*n* = 10)	*p*
Age (years)	41.65 ± 8.89	37 ± 13.90	0.25
Height (cm)	175 ± 10.27	179 ± 10.34	0.30
Weight (kg)	77.96 ± 19.08	74.2 ± 11.72	0.57
BMI (kg/m^2^)	25.14 ± 3.78	23 ± 1.84	0.10
Female/Male (*n*)	14/9	6/4	

Basic parametric data are presented as mean ± standard deviation for parametric data, categorical data are presented as numbers. Abbreviations: pwMS—people with multiple sclerosis; HC—healthy controls; BMI—body mass index; *n*—number of subjects according to gender.

**Table 2:** The differences in monocyte markers expression in people with MS and healthy controls.

		% of CD40^+^ of Total Monocytes	% of CD40^+^ CD14^+^CD16^++^	MFI of CD40 in CD14^+^CD16^++^	% CD40^+^ CD14^++^CD16^−^	MFI of CD40 in CD14^++^CD16^−^	MFI of CD192 in CD14^++^CD16^−^
All pwMS (N = 23)	M	71.85	91.55	3976.09	74.00	1904.00	68,443.67
	SD	10.62	9.42	1776.75	11.58	719.04	11,411.64
HC (N = 10)	M	59.50	87.59	3336.56	53.26	1180.03	82,983.35
	SD	13.72	9.51	700.70	16.83	701.25	8768.82
pwMS MEP Altered (N = 15)	M	71.59	92.03	3957.61	72.96	1954.22	67,545.47
	SD	11.19	7.25	1757.29	13.39	859.98	9819.92
pwMS MEP Normal (N = 8)	M	72.35	90.71	4008.45	75.96	1809.71	70,127.81
	SD	10.18	12.94	1932.27	7.49	360.83	14,541.25
All pwMS vs. HC	t	−2.8	1.09	−1.90	−4.11	−2.46	3.58
	df	31	31	29	32	30	32
	*p*	0.008 **	0.280	0.283	0.0002 ***	0.019 *	0.001 **
pwMS MEP Altered vs. HC	t	−2.41	−1.32	−1.105	−3.25	−2.18	4.01
	df	16	16	23	24	22	20
	*p*	0.023 *	0.203	0.303	0.003 **	0.04 *	0.0005 ***
pwMS MEP Normal vs. HC	t	−2.2	−0.59	−1.25	−3.52	−2.25	2.32
	df	16	12	17	17	15	11
	*p*	0.042 *	0.563	0.32	0.002 **	0.040 *	0.033 *
pwMS MEP Altered vs. pwMS MEP Normal	t	0.16	−0.31	0.063	0.58	−0.45	0.50
	df	22	21	21	22	22	22
	*p*	0.876	0.759	0.95	0.565	0.656	0.616

Abbreviations: pwMS—people with multiple sclerosis; HC—healthy control; pwMS MEP Altered—pwMS with altered MEP findings (prolonged MEP latency or absent MEP); pwMS MEP Normal—pwMS with normal MEP findings; MEP—motor-evoked potential; %—percentage; MFI—Median fluorescence intensity; M—arithmetic mean; SD—standard deviation; df—degree of freedom; t—*t* test. * *p* < 0.05; ** *p* < 0.01; *** *p* < 0.001.

## Data Availability

Further information regarding the resources and data availability should be directed to the corresponding author.
